# Supplementary material for: A Likelihood Approach for Real-Time Calibration of Stochastic Compartmental Epidemic Models
Source: PLoS Comput Biol. 2017 Jan 17;13(1):e1005257. doi: 10.1371/journal.pcbi.1005257 (PMC5240920; doi:10.1371/journal.pcbi.1005257)
Supplement: S1 File — (TAR.GZ) [file pcbi.1005257.s014.tar.gz › HSPH_Online-SI-Revision/EnKF07/n1-mild/n1-mild_EnKF2_table4.pdf]

1	newly 3 weeks spec	6.	21.12	{0., 65.}	344.333	0.925638	newly 3 weeks cumul	40.	126.39	{12., 394.}	258.675	0.402113	newly 5 weeks spec	2.	7.59	{0., 11.}	399.5	0.920279	newly 5 weeks cumul	43.	146.67	{12., 526.}	282.581	0.407013
2	newly 3 weeks spec	3.	12.51	{0., 21.}	417.	0.886244	newly 3 weeks cumul	16.	97.8	{6., 301.}	545.75	0.543006	newly 5 weeks spec	4.	3.6	{0., 8.}	144.5	1.2626	newly 5 weeks cumul	23.	107.51	{6., 313.}	410.043	0.508917
3	newly 3 weeks spec	13.	12.45	{0., 18.}	125.462	0.924119	newly 3 weeks cumul	35.	83.76	{7., 162.}	193.657	0.447928	newly 5 weeks spec	6.	3.6	{0., 7.}	114.667	1.22041	newly 5 weeks cumul	61.	94.01	{9., 199.}	138.016	0.519446
4	newly 3 weeks spec	9.	23.61	{1., 87.}	220.111	0.492056	newly 3 weeks cumul	30.	96.76	{16., 226.}	240.667	0.433735	newly 5 weeks spec	8.	13.51	{0., 34.}	176.375	0.838563	newly 5 weeks cumul	45.	128.49	{18., 364.}	208.467	0.378127
5	newly 3 weeks spec	10.	18.9	{0., 52.}	187.	0.672107	newly 3 weeks cumul	42.	118.21	{20., 195.}	206.167	0.295135	newly 5 weeks spec	5.	7.49	{0., 11.}	182.2	1.0683	newly 5 weeks cumul	52.	137.11	{21., 282.}	193.788	0.290723
6	newly 3 weeks spec	20.	13.93	{0., 26.}	116.85	1.18155	newly 3 weeks cumul	66.	87.65	{5., 184.}	133.682	0.558864	newly 5 weeks spec	12.	5.96	{0., 9.}	110.167	1.42676	newly 5 weeks cumul	97.	102.77	{8., 248.}	122.402	0.637788
7	newly 3 weeks spec	20.	7.07	{0., 17.}	98.25	1.58189	newly 3 weeks cumul	60.	116.43	{9., 189.}	160.617	0.439601	newly 5 weeks spec	5.	1.08	{0., 3.}	99.6	1.46593	newly 5 weeks cumul	86.	120.5	{9., 198.}	125.116	0.49048
8	newly 3 weeks spec	10.	13.61	{0., 25.}	151.7	0.762135	newly 3 weeks cumul	44.	52.72	{10., 71.}	111.	0.450045	newly 5 weeks spec	12.	7.99	{0., 16.}	126.75	1.44705	newly 5 weeks cumul	74.	71.55	{10., 156.}	113.635	0.601316
9	newly 3 weeks spec	13.	13.65	{0., 60.}	141.308	1.04611	newly 3 weeks cumul	57.	85.31	{5., 172.}	136.439	0.503599	newly 5 weeks spec	5.	4.23	{0., 15.}	135.8	1.29764	newly 5 weeks cumul	72.	96.89	{5., 287.}	131.208	0.558037
10	newly 3 weeks spec	5.	8.98	{0., 16.}	147.2	0.54962	newly 3 weeks cumul	23.	85.64	{14., 129.}	288.087	0.544503	newly 5 weeks spec	1.	2.93	{0., 9.}	329.	0.899402	newly 5 weeks cumul	26.	93.59	{14., 149.}	276.731	0.534853
11	newly 3 weeks spec	6.	15.53	{0., 23.}	253.833	0.803832	newly 3 weeks cumul	21.	132.46	{4., 220.}	557.048	0.650618	newly 5 weeks spec	11.	4.38	{0., 5.}	111.091	1.57711	newly 5 weeks cumul	43.	144.66	{4., 324.}	269.581	0.483491
12	newly 3 weeks spec	9.	11.55	{0., 10.}	168.333	0.988078	newly 3 weeks cumul	42.	53.37	{8., 57.}	107.881	0.398925	newly 5 weeks spec	9.	7.54	{0., 4.}	158.444	1.60834	newly 5 weeks cumul	57.	71.07	{8., 76.}	125.667	0.496894
13	newly 3 weeks spec	6.	14.69	{0., 20.}	282.5	1.25426	newly 3 weeks cumul	20.	94.66	{7., 136.}	397.7	0.47068	newly 5 weeks spec	0.1	5.15	{0., 9.}	5206.	0.422639	newly 5 weeks cumul	21.	109.21	{8., 136.}	444.905	0.478008
14	newly 3 weeks spec	4.	9.66	{0., 17.}	225.	0.821912	newly 3 weeks cumul	23.	65.13	{6., 119.}	209.348	0.413059	newly 5 weeks spec	3.	3.77	{0., 6.}	143.	0.889328	newly 5 weeks cumul	29.	75.31	{7., 145.}	187.966	0.393092
15	newly 3 weeks spec	19.	8.71	{0., 22.}	96.1579	1.28428	newly 3 weeks cumul	57.	116.52	{10., 146.}	161.649	0.379685	newly 5 weeks spec	13.	2.12	{0., 6.}	92.6154	1.71573	newly 5 weeks cumul	84.	123.01	{11., 182.}	130.512	0.4617
16	newly 3 weeks spec	24.	15.15	{0., 14.}	109.958	0.897704	newly 3 weeks cumul	51.	62.61	{8., 46.}	111.706	0.394871	newly 5 weeks spec	13.	8.33	{0., 10.}	122.538	1.25377	newly 5 weeks cumul	79.	81.84	{11., 58.}	115.013	0.492968
17	newly 3 weeks spec	19.	16.9	{0., 43.}	136.211	1.11613	newly 3 weeks cumul	42.	176.51	{9., 846.}	374.548	0.480023	newly 5 weeks spec	16.	1.64	{0., 4.}	92.	1.79254	newly 5 weeks cumul	69.	183.75	{10., 916.}	255.203	0.573096
18	newly 3 weeks spec	17.	14.34	{0., 19.}	124.706	1.06867	newly 3 weeks cumul	55.	93.25	{2., 172.}	155.182	0.613354	newly 5 weeks spec	17.	7.38	{0., 7.}	118.353	1.7025	newly 5 weeks cumul	88.	110.44	{2., 267.}	134.5	0.730336
19	newly 3 weeks spec	22.	23.17	{0., 75.}	145.409	1.21213	newly 3 weeks cumul	64.	143.1	{4., 443.}	209.219	0.583612	newly 5 weeks spec	12.	8.45	{0., 18.}	131.917	1.51713	newly 5 weeks cumul	88.	165.77	{4., 662.}	188.761	0.657029
20	newly 3 weeks spec	10.	12.28	{0., 16.}	144.	0.935573	newly 3 weeks cumul	38.	121.53	{13., 198.}	246.184	0.37301	newly 5 weeks spec	11.	2.82	{0., 4.}	103.273	1.65209	newly 5 weeks cumul	53.	131.99	{15., 204.}	188.509	0.350208
21	newly 3 weeks spec	11.	17.32	{0., 38.}	172.909	0.88956	newly 3 weeks cumul	47.	139.99	{12., 519.}	247.766	0.40236	newly 5 weeks spec	5.	8.02	{0., 12.}	192.4	1.0427	newly 5 weeks cumul	63.	160.06	{15., 636.}	215.429	0.421046
22	newly 3 weeks spec	16.	6.93	{0., 11.}	104.563	1.48509	newly 3 weeks cumul	38.	105.75	{12., 252.}	219.237	0.381925	newly 5 weeks spec	16.	1.21	{0., 3.}	96.3125	1.89861	newly 5 weeks cumul	74.	109.46	{12., 253.}	126.892	0.43413
23	newly 3 weeks spec	14.	21.48	{0., 20.}	171.286	0.853485	newly 3 weeks cumul	59.	103.28	{14., 116.}	133.627	0.359604	newly 5 weeks spec	8.	5.99	{0., 10.}	124.375	1.26317	newly 5 weeks cumul	90.	121.39	{20., 133.}	117.167	0.411773
24	newly 3 weeks spec	16.	8.68	{0., 14.}	119.	1.5584	newly 3 weeks cumul	68.	51.96	{3., 89.}	106.382	0.707482	newly 5 weeks spec	13.	1.75	{0., 6.}	93.6154	1.79715	newly 5 weeks cumul	98.	57.32	{3., 103.}	101.163	0.822779
25	newly 3 weeks spec	13.	17.72	{2., 28.}	114.308	0.464869	newly 3 weeks cumul	29.	127.36	{18., 344.}	355.034	0.395225	newly 5 weeks spec	8.	8.65	{0., 10.}	131.125	0.81197	newly 5 weeks cumul	44.	148.67	{23., 637.}	262.25	0.34459
26	newly 3 weeks spec	5.	14.07	{0., 16.}	265.	0.810854	newly 3 weeks cumul	24.	72.33	{7., 92.}	240.708	0.418053	newly 5 weeks spec	1.	10.17	{0., 12.}	1001.	0.851153	newly 5 weeks cumul	34.	94.05	{7., 97.}	221.147	0.41569
27	newly 3 weeks spec	19.	7.07	{0., 9.}	108.158	1.64028	newly 3 weeks cumul	39.	80.79	{4., 91.}	170.59	0.424071	newly 5 weeks spec	10.	2.5	{0., 3.}	106.8	1.74177	newly 5 weeks cumul	66.	87.07	{5., 103.}	132.348	0.551865
28	newly 3 weeks spec	12.	4.47	{0., 10.}	83.25	1.15767	newly 3 weeks cumul	35.	49.28	{6., 81.}	93.2571	0.38742	newly 5 weeks spec	8.	1.06	{0., 3.}	91.25	1.56744	newly 5 weeks cumul	50.	52.57	{6., 85.}	78.1	0.437324
29	newly 3 weeks spec	13.	4.74	{0., 5.}	107.692	1.48724	newly 3 weeks cumul	47.	46.09	{2., 43.}	124.702	0.673261	newly 5 weeks spec	5.	1.03	{0., 2.}	102.2	1.49744	newly 5 weeks cumul	61.	49.34	{2., 44.}	118.164	0.759248
30	newly 3 weeks spec	22.	24.88	{0., 47.}	161.	1.36619	newly 3 weeks cumul	51.	121.2	{5., 459.}	207.882	0.499391	newly 5 weeks spec	11.	11.43	{0., 43.}	161.545	1.58571	newly 5 weeks cumul	78.	149.78	{5., 649.}	182.821	0.579926
31	newly 3 weeks spec	4.	20.03	{1., 50.}	435.75	0.590344	newly 3 weeks cumul	23.	88.73	{13., 176.}	302.391	0.436135	newly 5 weeks spec	0.1	10.78	{0., 30.}	10732.	1.30575	newly 5 weeks cumul	29.	114.04	{16., 248.}	310.621	0.429573
32	newly 3 weeks spec	21.	12.5	{0., 30.}	114.381	1.44924	newly 3 weeks cumul	44.	101.14	{9., 278.}	211.909	0.497891	newly 5 weeks spec	15.	3.48	{0., 3.}	102.933	1.84507	newly 5 weeks cumul	72.	110.47	{9., 290.}	159.764	0.602156
33	newly 3 weeks spec	13.	12.57	{0., 12.}	124.385	0.792834	newly 3 weeks cumul	55.	49.22	{8., 84.}	91.9636	0.484341	newly 5 weeks spec	7.	5.6	{0., 5.}	126.857	1.09313	newly 5 weeks cumul	72.	63.34	{10., 93.}	98.6944	0.528994
34	newly 3 weeks spec	12.	20.9	{0., 51.}	159.667	0.631245	newly 3 weeks cumul	36.	156.43	{15., 540.}	360.528	0.481721	newly 5 weeks spec	15.	7.22	{0., 16.}	101.2	1.27279	newly 5 weeks cumul	60.	175.58	{16., 569.}	232.967	0.433912
35	newly 3 weeks spec	15.	11.79	{0., 18.}	103.8	0.938713	newly 3 weeks cumul	76.	95.82	{15., 108.}	104.105	0.388606	newly 5 weeks spec	15.	7.46	{0., 6.}	117.333	1.5172	newly 5 weeks cumul	115.	113.13	{17., 183.}	101.157	0.499596
36	newly 3 weeks spec	2.	23.39	{0., 52.}	1107.5	0.798607	newly 3 weeks cumul	28.	171.44	{14., 489.}	529.714	0.54443	newly 5 weeks spec	0.1	6.75	{0., 26.}	6742.	0.89811	newly 5 weeks cumul	28.	190.5	{16., 722.}	595.143	0.573871
37	newly 3 weeks spec	11.	12.06	{0., 32.}	107.091	0.648664	newly 3 weeks cumul	35.	72.97	{7., 155.}	160.314	0.416659	newly 5 weeks spec	8.	4.14	{0., 10.}	91.5	1.04404	newly 5 weeks cumul	53.	83.55	{7., 172.}	125.377	0.440986
38	newly 3 weeks spec	9.	21.21	{0., 57.}	252.556	1.00144	newly 3 weeks cumul	41.	113.12	{5., 305.}	239.171	0.494454	newly 5 weeks spec	5.	7.02	{0., 20.}	194.8	1.31058	newly 5 weeks cumul	58.	132.11	{5., 341.}	208.845	0.555976
39	newly 3 weeks spec	13.	13.35	{0., 19.}	127.308	0.834349	newly 3 weeks cumul	39.	76.6	{5., 140.}	173.179	0.463099	newly 5 weeks spec	4.	4.89	{0., 9.}	150.75	0.947088	newly 5 weeks cumul	49.	88.81	{6., 172.}	159.735	0.463158
40	newly 3 weeks spec	11.	15.86	{0., 14.}	165.636	0.812559	newly 3 weeks cumul	36.	69.73	{4., 55.}	159.306	0.393541	newly 5 weeks spec	1.	6.98	{0., 8.}	694.	0.754645	newly 5 weeks cumul	43.	86.78	{5., 96.}	174.	0.428544
41	newly 3 weeks spec	9.	10.1	{0., 14.}	130.222	0.84682	newly 3 weeks cumul	33.	62.27	{6., 69.}	145.364	0.347858	newly 5 weeks spec	5.	5.01	{0., 9.}	142.2	1.11004	newly 5 weeks cumul	42.	74.53	{7., 96.}	143.5	0.386446
42	newly 3 weeks spec	0.1	11.9	{0., 30.}	11842.	1.46482	newly 3 weeks cumul	0.1	87.68	{10., 229.}	87582.	2.52193	newly 5 weeks spec	0.1	7.26	{0., 7.}	7258.	0.821782	newly 5 weeks cumul	0.1	103.51	{11., 277.}	103412.	2.58366
43	newly 3 weeks spec	2.	20.37	{0., 43.}	971.5	0.670641	newly 3 weeks cumul	19.	88.37	{5., 261.}	392.263	0.440791	newly 5 weeks spec	2.	8.6	{0., 14.}	463.	1.01242	newly 5 weeks cumul	24.	111.47	{6., 337.}	394.125	0.41538
44	newly 3 weeks spec	2.	9.94	{0., 16.}	445.	0.658882	newly 3 weeks cumul	12.	48.13	{5., 60.}	321.25	0.510271	newly 5 weeks spec	0.1	4.26	{0., 7.}	4262.	0.761279	newly 5 weeks cumul	14.	58.91	{5., 71.}	342.214	0.508976
45	newly 3 weeks spec	13.	19.27	{0., 38.}	167.923	0.98594	newly 3 weeks cumul	46.	139.62	{8., 494.}	252.565	0.474149	newly 5 weeks spec	7.	5.36	{0., 7.}	129.714	1.22352	newly 5 weeks cumul	63.	155.24	{10., 606.}	211.556	0.504273
46	newly 3 weeks spec	11.	18.54	{0., 14.}	217.636	1.44129	newly 3 weeks cumul	54.	131.19	{3., 506.}	221.87	0.580034	newly 5 weeks spec	9.	7.61	{0., 7.}	152.111	1.58874	newly 5 weeks cumul	77.	151.	{3., 525.}	193.584	0.637829
47	newly 3 weeks spec	14.	5.74	{0., 12.}	98.8571	1.36104	newly 3 weeks cumul	32.	67.64	{3., 93.}	164.75	0.405344	newly 5 weeks spec	10.	0.7	{0., 1.}	93.2	1.7751	newly 5 weeks cumul	50.	70.1	{3., 112.}	121.76	0.473944
48	newly 3 weeks spec	9.	10.59	{0., 12.}	129.889	0.731262	newly 3 weeks cumul	23.	70.57	{9., 63.}	239.696	0.356769	newly 5 weeks spec	3.	4.65	{0., 4.}	197.667	1.07949	newly 5 weeks cumul	34.	81.98	{10., 70.}	188.882	0.348218
49	newly 3 weeks spec	8.	8.79	{0., 39.}	146.625	1.16566	newly 3 weeks cumul	37.	41.95	{7., 101.}	91.4324	0.430912	newly 5 weeks spec	3.	5.42	{0., 19.}	233.333	1.25224	newly 5 weeks cumul	43.	54.32	{8., 180.}	111.07	0.477619
50	newly 3 weeks spec	19.	26.68	{0., 65.}	181.474	1.20718	newly 3 weeks cumul	60.	148.41	{6., 651.}	245.25	0.590225	newly 5 weeks spec	13.	4.91	{0., 4.}	111.769	1.66125	newly 5 weeks cumul	91.	165.68	{6., 811.}	199.187	0.700875

1	newly 51 weeks cumul	116.	157.16	{12., 526.}	141.293	0.57587
2	newly 47 weeks cumul	28.	112.24	{6., 313.}	352.	0.515182
3	newly 56 weeks cumul	77.	98.37	{9., 242.}	123.727	0.567719
4	newly 50 weeks cumul	300.	156.92	{21., 474.}	84.34	0.664514
5	newly 52 weeks cumul	70.	155.27	{25., 451.}	169.9	0.316593
6	newly 56 weeks cumul	201.	114.3	{8., 272.}	97.4826	0.835998
7	newly 55 weeks cumul	126.	121.44	{9., 198.}	104.683	0.588457
8	newly 47 weeks cumul	324.	93.23	{12., 206.}	91.6512	1.07852
9	newly 46 weeks cumul	74.	106.69	{5., 293.}	141.338	0.577188
10	newly 47 weeks cumul	26.	101.53	{14., 160.}	307.038	0.558287
11	newly 46 weeks cumul	315.	150.51	{4., 391.}	91.8	0.876138
12	newly 47 weeks cumul	206.	80.12	{8., 104.}	97.2524	0.948938
13	newly 48 weeks cumul	26.	118.04	{8., 136.}	385.769	0.457525
14	newly 52 weeks cumul	29.	82.87	{7., 153.}	212.931	0.415942
15	newly 52 weeks cumul	175.	127.72	{11., 233.}	99.2457	0.674626
16	newly 50 weeks cumul	230.	109.74	{14., 70.}	102.061	0.846662
17	newly 53 weeks cumul	203.	185.39	{10., 933.}	131.768	0.881611
18	newly 46 weeks cumul	176.	121.41	{2., 303.}	109.597	0.950441
19	newly 53 weeks cumul	114.	180.97	{5., 799.}	170.763	0.720195
20	newly 50 weeks cumul	69.	134.47	{15., 205.}	151.957	0.372019
21	newly 50 weeks cumul	68.	176.89	{17., 659.}	221.721	0.428505
22	newly 52 weeks cumul	332.	110.86	{12., 253.}	86.8434	0.897183
23	newly 47 weeks cumul	150.	135.26	{21., 162.}	104.36	0.55666
24	newly 49 weeks cumul	197.	59.36	{3., 103.}	91.8782	1.06544
25	newly 46 weeks cumul	191.	170.73	{25., 683.}	105.094	0.566311
26	newly 49 weeks cumul	44.	135.99	{7., 219.}	252.205	0.433208
27	newly 46 weeks cumul	107.	92.53	{5., 103.}	114.813	0.706766
28	newly 52 weeks cumul	232.	54.86	{6., 98.}	81.8534	0.9424
29	newly 49 weeks cumul	61.	51.51	{2., 47.}	119.361	0.755009
30	newly 53 weeks cumul	111.	171.77	{6., 696.}	162.748	0.674618
31	newly 51 weeks cumul	29.	142.48	{19., 445.}	406.276	0.491445
32	newly 49 weeks cumul	281.	118.44	{9., 306.}	93.2313	0.988841
33	newly 50 weeks cumul	85.	73.97	{12., 102.}	99.5647	0.545878
34	newly 49 weeks cumul	138.	192.52	{16., 576.}	132.13	0.555954
35	newly 51 weeks cumul	153.	125.8	{17., 240.}	100.013	0.587409
36	newly 53 weeks cumul	28.	208.2	{16., 789.}	658.071	0.604792
37	newly 49 weeks cumul	95.	90.35	{8., 208.}	98.3263	0.572859
38	newly 53 weeks cumul	65.	142.14	{5., 456.}	205.815	0.585923
39	newly 51 weeks cumul	78.	109.28	{6., 262.}	140.897	0.567238
40	newly 54 weeks cumul	43.	106.	{5., 112.}	213.535	0.440373
41	newly 55 weeks cumul	48.	94.55	{7., 142.}	165.479	0.421405
42	newly 54 weeks cumul	0.1	125.51	{11., 368.}	125412.	2.62634
43	newly 56 weeks cumul	27.	125.95	{6., 556.}	398.481	0.41046
44	newly 48 weeks cumul	17.	66.72	{5., 86.}	317.647	0.491593
45	newly 46 weeks cumul	86.	160.3	{10., 629.}	171.14	0.560616
46	newly 53 weeks cumul	299.	166.02	{3., 562.}	107.873	1.0403
47	newly 51 weeks cumul	260.	70.55	{3., 112.}	89.45	1.02713
48	newly 46 weeks cumul	83.	91.3	{10., 78.}	119.229	0.534943
49	newly 50 weeks cumul	190.	65.03	{8., 235.}	83.5421	0.917377
50	newly 47 weeks cumul	148.	169.69	{6., 828.}	149.628	0.837626
